# Assessment of antiproliferative and antiplasmodial activities of five selected Apocynaceae species

**DOI:** 10.1186/1472-6882-11-3

**Published:** 2011-01-14

**Authors:** Siu Kuin Wong, Yau Yan Lim, Noor Rain Abdullah, Fariza Juliana Nordin

**Affiliations:** 1 School of Science, Monash University Sunway Campus, Bandar Sunway, 46150 Petaling Jaya, Selangor, Malaysia; 2 Herbal Medicine Research Centre, Institute for Medical Research, 50588 Kuala Lumpur, Malaysia

## Abstract

**Background:**

Studies have shown that the barks and roots of some Apocynaceae species have anticancer and antimalarial properties. In this study, leaf extracts of five selected species of Apocynaceae used in traditional medicine (*Alstonia angustiloba*, *Calotropis gigantea*, *Dyera costulata*, *Kopsia fruticosa *and *Vallaris glabra*) were assessed for antiproliferative (APF) and antiplasmodial (APM) activities, and analysed for total alkaloid content (TAC), total phenolic content (TPC) and radical-scavenging activity (RSA). As *V. glabra *leaf extracts showed wide spectrum APF and APM activities, they were further screened for saponins, tannins, cardenolides and terpenoids.

**Methods:**

APF and APM activities were assessed using the sulphorhodamine B and lactate dehydrogenase assays, respectively. TAC, TPC and RSA were analysed using Dragendorff precipitation, Folin-Ciocalteu and DPPH assays, respectively. Screening for saponins, tannins, cardenolides and terpenoids were conducted using the frothing, ferric chloride, Kedde and vanillin-H_2_SO_4 _tests, respectively.

**Results:**

Leaf extracts of *A. angustiloba*, *C. gigantea *and *V. glabra *displayed positive APF activity. Dichloromethane (DCM) extract of *C. gigantea*, and DCM and DCM:MeOH extracts of *V. glabra *showed strong APF activity against all six human cancer cell lines tested. DCM extract of *A. angustiloba *was effective against three cancer cell lines. Against MCF-7 and MDA-MB-231 cell lines, DCM extract of *C. gigantea *was stronger than standard drugs of xanthorrhizol, curcumin and tamoxifen. All five species were effective against K1 strain of *Plasmodium falciparum *and three species (*C. gigantea, D. costulata and K. fruticosa*) were effective against 3D7 strain. Against K1 strain, all four extracts of *V. glabra *displayed effective APM activity. Extracts of *D. costulata *were effective against 3D7 strain. Selectivity index values of extracts of *A. angustiloba*, *C. gigantea *and *V. glabra *suggested that they are potentially safe for use to treat malaria. Extracts of *K. fruticosa *had the highest TAC while *D. costulata *had the highest TPC and RSA. Phytochemical screening of extracts of *V. glabra *also showed the presence of terpenoids, tannins and saponins.

**Conclusions:**

Leaf extracts of *C. gigantea *and *V. glabra *showed great promise as potential candidates for anticancer drugs as they inhibited the growth of all six cancer cell lines. Against K1 strain of *P. falciparum*, all four extracts of *V. glabra *displayed effective APM activity. The wide spectrum APF and APM activities of *V. glabra *are reported for the first time and this warrants further investigation into its bioactive compounds.

## Background

The family Apocynaceae consists of about 250 genera and 2000 species of tropical trees, shrubs and vines [1,2]. A characteristic feature of the family is that almost all species produce milky sap. Leaves are simple, opposite and whorled. Flowers are large and colourful.

In traditional medicine, Apocynaceae species are used to treat gastrointestinal ailments, fever, malaria, pain and diabetes [2]. Of the five species studied, roots and leaves of *Calotropis gigantea *(L.) Aiton are used to treat skin and liver diseases, leprosy, dysentery, worms, ulcers, tumours and earaches [3]. Its latex has been reported to have wound healing properties [4]. *Kopsia fruticosa *(Ker.) A. DC. is used to treat sore and syphilis, and has cholinergic effects [5]. Leaves and barks of *Dyera costulata *Hook have been used for treating fever, inflammation and pain [6]. Stems, leaves and latex of *Alstonia angustiloba *Miq. have been used for gynaecological problems and skin sores in Indonesia [7]. Leaves are externally applied to treat headache in Malaysia [8]. *Vallaris glabra *Kuntze is well known in Thailand because the scent of its flowers is similar to that of pandan leaves and aromatic rice [9]. Its use in traditional medicine has not been reported, and its bioactivity and phytochemistry have yet to be studied. Species of Apocynaceae have also been reported to possess anticancer properties [2,10]. Species having cytotoxic activity include those of *Allamanda *[11], *Alstonia *[12,13], *Cerbera *[14,15], *Nerium *[16,17], *Plumeria *[18] and *Tabernaemontana *[19]. Species of Apocynaceae, notably those of *Alstonia*, are also known to have antimalarial properties [20-22].

Prompted by the anticancer and antimalarial properties of Apocynaceae, leaf extracts of five selected species used in traditional medicine were assessed for antiproliferative (APF) activity against six human cancer cell lines and for antiplasmodial (APM) activity against two strains of *Plasmodium falciparum*. Their extracts were also analysed for total alkaloid content, total phenolic content and radical-scavenging activity. Having wide spectrum APF and APM activities, leaf extracts of *V. glabra *were further screened for saponins, tannins, cardenolides and terpenoids. Information from the screening will serve as a useful guide to further work on isolating compounds with APF and APM activities.

## Methods

### Plant materials

Species studied were *A. angustiloba*, *C. gigantea*, *D. costulata*, *K. fruticosa *and *V. glabra. *Leaf samples of *A. angustiloba*, *C. gigantea *and *V. glabra *were collected from Puchong (3°2'42"N; 101°37'12"E), Sunway (3°4'30"N; 101°36'8" E) and Kepong (3°12'14"N; 101°37'50"E) in Selangor, Malaysia, respectively. Those of *D. costulata *and *K. fruticosa *were collected from the Forest Research Institute Malaysia (3°14'6"N; 101°37'58"E). Identification of species was based on documented descriptions and illustrations [1,2]. With brief descriptions of their morphology and location of collection, the voucher specimens of these species (WSK01, WSK02, WSK03, WSK04 and WSK05, respectively) were deposited in the herbarium of Monash University Sunway Campus in Malaysia.

### Extraction of leaves

For crude extraction, fresh leaves of each species (40 g) were cut into small pieces and freeze-dried overnight. Dried samples were blended and extracted with 250 ml of methanol (MeOH) three times for 1 h each time. Samples were filtered and the solvent was removed using a rotary evaporator (Eyela). The dried crude extracts were stored at -20°C for further analysis. For sequential extraction, fresh leaves of each species (40 g) were freeze-dried, ground and extracted successively with hexane (HEX), dichloromethane (DCM), DCM:MeOH (1:1) and MeOH (HmbG Chemicals). For each solvent, the suspension of ground leaves in 250-300 ml of solvent was shaken for 1 h on the orbital shaker. After filtering, the samples were extracted two more times for each solvent. Solvents were removed with a rotary evaporator to obtain the dried extracts, which were stored at -20°C for further analysis.

### Antiproliferative activity

Antiproliferative (APF) activity of extracts (25 μg/ml) was initially screened for growth inhibitory activity against three human cancer cell lines (MCF-7, MDA-MB-231 and HeLa) using the sulphorhodamine B (SRB) assay [23]. Growth inhibitory activity with less than 50% cell growth was considered positive while that with more than 50% cell growth was considered negative. Extracts with positive growth inhibition were further tested against six human cancer lines (MCF-7, MDA-MB-231, HeLa, HT-29, SKOV-3 and HepG2) using six different extract concentrations. Human cancer cell lines were obtained from the American Type Culture Collection (ATCC, Manassas, VA, USA). The cells were seeded 24 h prior to treatment in 96-well plates at densities of 10,000-20,000 cells/well. Each cell line was designated one plate. Initial cell population of each cell lines prior to addition of extracts was determined by fixing with trichloroacetic acid (TCA) (Sigma). Extracts were dissolved in dimethyl sulphoxide (DMSO) (Sigma) and serially diluted from 8-25 μg/ml. Control cultures were treated with the same volume of DMSO. The concentration of DMSO was kept within 1% to avoid any interference with cell viability. After the addition of extracts, the plates were incubated for 48 h. After incubation, the cells were fixed with 50 μl of cold 50% TCA and incubated for 1 h at 4°C. The plates were then washed with tap water and air dried. Cells were stained with 100 μl of 0.4% SRB solution (Sigma) diluted with 1% acetic acid followed by incubation for 10 min at room temperature. Unbound dye was removed by washing with 1% acetic acid. Bound stain was then solubilised with 200 μl of 10 mM trizma base (Sigma). Absorbance of each well at 505 nm was obtained using a microplate reader. Dose-response curves were constructed to obtain GI_50 _or concentration of extract that causes growth inhibition (GI) of cells by 50%. GI_50 _was calculated using the formula [(T_z _- T_i_)/(T_c _- T_i_)] × 100 = 50 where T_z _is the absorbance of cells treated with extracts or drugs at the end of incubation, T_i _is the absorbance of cells prior to treatment with extracts or drugs and T_c _is the absorbance of untreated cells at the end of incubation [24]. IC_50 _or inhibition concentration at which there is a 50% reduction in cells was obtained using the formula T_z_/T_c _× 100 = 50. Activity is considered to be effective when GI_50 _value ≤20 μg/ml [25].

### Antiplasmodial activity

Antiplasmodial (APM) activity of extracts was assessed *in vitro *in human blood using the lactate dehydrogenase assay with slight modifications [26]. Chloroquine-resistant K1 and chloroquine-sensitive 3D7 strains of *Plasmodium falciparum *were tested. Standard drugs of artemisinin (Sigma) and mefloquine (Sigma) were used as positive controls. Extracts dissolved in DMSO (10 mg/ml) were diluted with deionised water to 320 μg/ml. The solution was serially diluted two-fold six times to give seven different concentrations. Aliquots of each concentration (10 μl) were transferred into 96-well microtiter plates. Parasitised red blood cell suspensions (1% parasitaemia, 190 μl) were added to each well. Parasitised and non-parasitised red blood cells were used as negative controls. The plates were incubated for 24 h at 37°C in a candle jar and were subsequently chilled at -20°C to lyse the red blood cells. The plates were then allowed to cool to room temperature, and 20 μl of blood suspension was dispensed into a new microtiter plate containing 100 μl MALSTAT™ reagent (Flow Inc.), and 20 μl of nitroblue tetrazolium (Sigma) and phenazine ethosulphate (Sigma) mixture. Absorbance was measured with a plate reader at 630 nm. Percentage inhibition at each concentration was determined and the mean of EC_50 _values of parasite viability was calculated using probit analysis. EC_50 _or effective concentration is the extract concentration that kills 50% of malaria parasites. Activity is effective if EC_50 _value ≤10 μg/ml [27]. The selectivity index (SI) for APM activity was calculated based on the ratio of cytotoxicity (IC_50_) on HepG2 and MCF-7 cells to APM activity (EC_50_) on chloroquine-resistant K1 strain.

### Analysis of TAC, TPC and RSA

#### Total alkaloid content

Total alkaloid content (TAC) of extracts was determined using the Dragendorff precipitation assay [28]. For each species, extracts (15 mg) were dissolved in 1 ml of distilled water that was acidified to pH 2.0-2.5 with 0.01 M HCl. Analysis was conducted in triplicate. Alkaloids were then precipitated with 0.4 ml of Dragendorff reagent. After washing with 0.5 ml of distilled water to remove traces of the reagent, the precipitate was later treated with 0.4 ml of 1% sodium sulphide, resulting in a brownish-black precipitate. Precipitates formed at each stage were recovered by centrifugation at 14,000 rpm for 1 min. The resulting precipitate was dissolved in 0.2 ml of concentrated nitric acid and diluted to 1 ml with distilled water. Addition of 2.5 ml of 3% thiourea to 0.5 ml aliquots of this solution resulted in a yellow colored complex. Absorbance was measured at 435 nm and TAC was expressed as boldine equivalent in milligram per gram of extract. The calibration equation for boldine (Sigma) was y = 1.068x (*R*^2 ^= 0.9959) where y is absorbance and × is mg/ml of boldine. Dragendorff reagent was prepared by dissolving 0.8 g of bismuth nitrate (Sigma) in 40 ml of distilled water and 10 ml of glacial acetic acid. The resulting solution was mixed with 20 ml of 40% potassium iodide.

#### Total phenolic content

Total phenolic content (TPC) of extracts was determined using the Folin-Ciocalteu (FC) assay [29-31]. Extracts (300 μl in triplicate) were introduced into test tubes followed by 1.5 ml of FC reagent (Fluka) at 10 times dilution and 1.2 ml of sodium carbonate (Fluka) at 7.5% w/v. The tubes were allowed to stand for 30 min in the dark before absorbance was measured at 765 nm. TPC was expressed as gallic acid (GA) equivalent in milligram per gram of extract. The calibration equation for GA (Fluka) was y = 0.0111x - 0.0148 (*R*^2 ^= 0.9998) where y is absorbance and × is mg/ml of GA.

#### Radical-scavenging activity

Radical-scavenging activity (RSA) of extracts was determined using the 1,1-diphenyl-2-picrylhydrazyl (DPPH) assay [29-31]. Different dilutions of extracts (1 ml in triplicate) were added to 2 ml of DPPH (Sigma). The concentration of DPPH used was 5.9 mg in 100 ml of methanol. Absorbance was measured at 517 nm after 30 min. RSA was calculated as IC_50_, the concentration of extract to scavenge 50% of the DPPH radical. RSA was then expressed as ascorbic acid equivalent antioxidant capacity (AEAC) using the equation: AEAC (mg ascorbic acid/g) = IC_50(ascorbate)_/IC_50(sample) _× 10^5^. IC_50 _of ascorbic acid used for calculation of AEAC was 0.00387 mg/ml.

### Phytochemical screening

Qualitative phytochemical screening for saponins, tannins, cardenolides and terpenoids from leaf extracts of *V. glabra *was carried out using standard phytochemical procedures [32,33]. The frothing test was used for saponins, the ferric chloride test for tannins, the Kedde test for cardenolides and the vanillin-H_2_SO_4 _test for terpenoids.

## Results and Discussion

### APF activity

Initial screening of leaf extracts of five Apocynaceae species against three human cancer cell lines (MCF-7, MDA-MB-231 and HeLa) showed that DCM, DCM:MeOH and MeOH extracts of *A. angustiloba*, *C. gigantea *and *V. glabra *had growth inhibitory activity (data not shown). DCM and DCM:MeOH extracts of *V. glabra*, and DCM extract of *C. gigantea *inhibited all three cancer cell lines. HEX and MeOH extracts of all three species did not show inhibition, with the exception of MeOH extract of *V. glabra*. Extracts of *D. costulata *and *K. fruticosa *did not show any APF activity.

Extracts of the three species were further tested against six human cancer cell lines (MCF-7, MDA-MB-231, HeLa, HT-29, SKOV-3 and HepG2). Results showed that DCM extract of *A. angustiloba *inhibited only MDA-MB-231, HeLa and SKOV-3 cell lines with GI_50 _values of 20, 20 and 16 μg/ml, respectively (Table 1). DCM and DCM:MeOH extracts of *C. gigantea *inhibited all cancer cell lines except for DCM:MEOH extract against MDA-MB-231. APF activity of DCM extract of *C. gigantea *was the strongest with GI_50 _values ranging from 1.3 to 3.3 μg/ml. Against MCF-7 and MDA-MB-231, GI_50 _of DCM extract of *C. gigantea *(1.9 and 1.3 μg/ml) was stronger than that of xanthorrhizol (11 and 8.7 μg/ml), curcumin (4.1 and 8.7 μg/ml) and tamoxifen (8.3 and 4.6 μg/ml), respectively. DCM and DCM:MeOH extracts of *V. glabra *inhibited all cell lines with GI_50 _values ranging from 7.5-12 μg/ml and 5.8-13 μg/ml, respectively. In addition, MeOH extract of *V. glabra *also inhibited the growth of MCF-7 and HepG2. Against MCF-7, GI_50 _of DCM and DCM:MeOH extracts of *V. glabra *(7.7 and 7.0 μg/ml) was stronger than xanthorrhizol (11 μg/ml) and comparable to tamoxifen (8.3 μg/ml), respectively.

**Table 1:** Antiproliferative activity of leaf extracts of three Apocynaceae species against six human cancer cell lines^a^

Species	^**c**^**Leaf extract**	^**b**^**GI**_**50 **_**(μg/ml)**					
		
		MCF-7	MDA-MB-231	HeLa	HT-29	SKOV-3	HepG2
*Alstonia angustiloba*	HEX	-	-	-	-	-	-
	DCM	-	20 ± 1.7	20 ± 1.1	-	16 ± 1.4	-
	DCM:MeOH	-	-	-	-	-	-
	MeOH	-	-	-	-	-	-
*Calotropis gigantea*	HEX	-	-	-	-	-	-
	DCM	1.9 ± 0.2	1.3 ± 0.3	2.5 ± 0.5	3.3 ± 0.2	2.5 ± 0.2	1.8 ± 1.7
	DCM:MeOH	13 ± 0.3	-	15 ± 1.0	24 ± 0.7	20 ± 2.3	16 ± 3.5
	MeOH	-	-	-	-	-	-
*Vallaris glabra*	HEX	-	-	-	-	-	-
	DCM	7.7 ± 1.3	12 ± 2.0	9.8 ± 1.5	9.3 ± 2.0	7.5 ± 4.5	7.6 ± 0.2
	DCM:MeOH	7.0 ± 2.5	13 ± 6.3	8.5 ± 2.9	12 ± 1.2	7.7 ± 2.4	5.8 ± 1.2
	MeOH	16 ± 2.1	-	-	-	-	19 ± 0.9
^d^**Standard drug**							
Xanthorrhizol		11 ± 0.7	8.7 ± 0.8				
Curcumin		4.1 ± 0.9	8.7 ± 0.8				
Tamoxifen		8.3 ± 0.6	4.6 ± 0.5				

^a ^Initial screening showed that extracts of *Dyera costulata *and *Kopsia fruticosa *did not show any APF activity.

^b ^GI_50 _(μg/ml) is growth inhibition (GI) of cancer cell lines by 50%. Inhibition is not effective (-) with values >20 μg/ml. MCF-7 and MDA-MB-231, HeLa and SKOV-3, HT-29, and HepG2 are human breast, cervical, colon and liver cancer cell lines, respectively.

^c ^HEX, hexane; DCM, dichloromethane; MeOH, methanol.

^d ^Data on standard drugs of xanthorrhizol, curcumin and tamoxifen against MCF-7 and MDA-MD-231 cell lines are from an earlier publication [24].

To the best of our knowledge, this study represents the first report of cytotoxic activity from DCM leaf extract of *A. angustiloba *and DCM and DCM:MeOH extracts of *V. glabra*. Earlier studies have reported cytotoxic activity from the root bark of *Alstonia macrophylla *Wall. ex G. Don [12] and the stem bark of *Alstonia scholaris *R. Br. [13]. A recent study on *Vallaris solanacea *(Roth) Kuntz has reported potent cell growth inhibitory activity of cardenolide glycosides isolated from the plant [34]. The finding of strong APF activity from DCM and DCM:MeOH extracts of *C. gigantea *from this study is supported by an earlier report that DCM leaf extracts of *C. gigantea *had strong inhibitory activity against KB, BC and NCI-H187 cancer cell lines [35]. Ethanol root extracts of *C. gigantea *were also reported to be cytotoxic to K562 and SGC-7901 human cell lines [36].

### APM activity

Against chloroquine-resistant K1 strain of *P. falciparum*, leaves of *V. glabra *were most effective as all four extracts had APM activity with EC_50 _less than 10 μg/ml (Table 2). DCM extract was the strongest with EC_50 _of 0.85 μg/ml. Three extracts of *A. angustiloba*, and two extracts of *C. gigantea*, *D. costulata *and *K. fruticosa *showed APM activity. It should be noted that DCM:MeOH extracts of all five species displayed APM activity with *A. angustiloba *having the strongest activity (EC_50 _of 0.46 μg/ml).

**Table 2:** Antiplasmodial (APM) activity and selectivity index (SI) of leaf extracts of Apocynaceae species

Species	^**c**^**Leaf extract**	^**a**^**EC**_**50 **_**(μg/ml) of APM activity**		^**b**^**IC**_**50 **_**(μg/ml) of APF activity**		^**b**^**SI of APM activity**	
		
		K1	3D7	HepG2	MCF-7	HepG2	MCF-7
*Alstonia angustiloba*	HEX	7.81	-				
	DCM	-	-				
	DCM:MeOH	0.46	-	>25.0	>25.0	NC	NC
	MeOH	6.46	-				
*Calotropis gigantea*	HEX	5.82	-				
	DCM	-	-				
	DCM:MeOH	0.97	3.29	>25.0	16.8	NC	17.3
	MeOH	-	-				
*Dyera costulata*	HEX	-	-				
	DCM	-	8.31				
	DCM:MeOH	7.52	2.13				
	MeOH	7.74	3.56				
*Kopsia fruticosa*	HEX	-	-				
	DCM	-	7.14				
	DCM:MeOH	4.35	-				
	MeOH	1.01	-				
*Vallaris glabra*	HEX	1.00	-	>25.0	>25.0	NC	NC
	DCM	0.85	-	>25.0	12.0	NC	14.1
	DCM:MeOH	8.45	-				
	MeOH	8.42	-				
^d^**Standard drug**							
Artemisinin		0.001	0.001				
Mefloquine		0.008	0.018				

^a ^EC_50 _or effective concentration (μg/ml) is the extract concentration that kills 50% of malaria parasites. Activity is not effective (-) if EC_50 _value >10 μg/ml. K1 and 3D7 are chloroquine-resistant and chloroquine-sensitive strains of *Plasmodium falciparum*, respectively.

^b ^IC_50 _of antiproliferative (APF) activity and SI of APM activity against HepG2 and MCF-7 cells were calculated only for the most potent extracts against K1 strain with EC_50 _≤1.00 μg/ml. NC, non-cytotoxic with SI values >25.0.

^c ^HEX, hexane; MeOH, methanol; DCM, dichloromethane.

^d ^Standard drugs of artemisinin and mefloquine are used as positive controls.

Against chloroquine-sensitive 3D7 strain of *P. falciparum*, extracts of *A. angustiloba *and *V. glabra *showed no activity. Extracts of *D. costulata *were the exception in that DCM, DCM:MeOH and MeOH extracts showed positive APM activity with EC_50 _of 8.31, 2.13 and 3.56 μg/ml, respectively. Generally, extracts were less effective against 3D7 strain.

This finding complements an earlier report that alkaloids from the extracts of *Alstonia *species were effective against chloroquine-resistant K1 strain but not against chloroquine-sensitive T9-96 strain [22]. Previous studies on the APM activity of Apocynaceae were focused on *Alstonia *species [21,22]. This study is the first to report the APM activity of leaf extracts of *D. costulata*, *C. gigantea*, *K. fruticosa *and *V. glabra*. A notable finding is the APM activity of *V. glabra *against K1 strain in all solvent fractions.

### Selectivity index

The selectivity index (SI) for APM activity indicates the safety of an extract to be used for antimalarial therapy [37]. The index was calculated based on the ratio of cytotoxicity (IC_50_) on HepG2 and MCF-7 cells to APM activity (EC_50_). The SI was calculated for the most potent extracts against K1 strain of *P. falciparum *with EC_50 _≤1.00 μg/ml. They were DCM:MeOH extracts of *A. angustiloba *(0.46 μg/ml) and *C. gigantea *(0.97 μg/ml), and HEX and DCM extracts of *V. glabra *(1.00 and 0.85 μg/ml), respectively (Table 2). Against HepG2 cells, IC_50 _values were all >25.0 μg/ml, and against MCF-7 cells, IC_50 _values were >25.0, 16.8, >25.0 and 12.0 μg/ml, respectively.

Against HepG2 cells, HEX and DCM extracts of *V. glabra*, and DCM:MeOH extracts of *A. angustiloba *and *C. gigantea *were non-cytotoxic with SI values >25.0 (Table 2). Against MCF-7 cells, the DCM extract of *V. glabra *and the DCM:MeOH extract of *C. gigantea *had SI values of 14.1 and 17.3, respectively. The HEX extract of *V. glabra *and the DCM:MeOH extract of *A. angustiloba *were non-cytotoxic with SI values >25.0.

Recently, a study on the antimalarial and cytotoxic activity of plants in the Democratic Republic of Congo considered SI values of extracts >10 as high [38]. With SI values relatively higher than 10, the extracts of *A. angustiloba*, *C. gigantea *and *V. glabra *are potentially safe for use to treat malaria.

### Analysis of TAC, TPC and RSA

Of the five species analysed, MeOH crude and DCM extracts of *K. fruticosa *had the highest TAC (100 and 129 mg BE/g of extract), respectively (Table 3). Other species with high TAC were *D. costulata *and *A. angustiloba *with MeOH crude and DCM:MeOH extracts having values in the range 58-68 and 23-58 mg BE/g of extract, respectively. Based on TAC, the ranking of species was: *K. fruticosa *>*D. costulata *>*A. angustiloba *>*C. gigantea *≈ *V. glabra*. Extracts of *D. costulata *had the highest TPC and strongest RSA. MeOH crude, DCM:MeOH and MeOH extracts yielded TPC values of 319, 354 and 279 mg GAE/g of extract, and RSA values of 377, 349 and 278 mg AA/g of extract, respectively. Based on TPC and RSA, the ranking of species was: *D. costulata *>*V. glabra *>*K. fruticosa *>*A. angustiloba *>*C. gigantea*. There is a strong correlation between TPC and RSA of extracts (*R*^2 ^= 0.955) but not with TAC (*R*^2 ^= 0.112). Correlation of results of phytochemical analysis with APF and APM activities remains unclear. Extracts of *C. gigantea *and *V. glabra *showed strong APF activity. The former had low TAC and TPC, while the latter had low TAC but high TPC. Extracts of *V. glabra *and *D. costulata *were effective against K1 and 3D7 strains of *P. falciparum*, respectively. The former had low TAC but high TPC, while the latter had high TAC and TPC.

**Table 3:** Phytochemical analysis of total alkaloid content, total phenolic content and radical-scavenging activity of leaf extracts of Apocynaceae species

Species	MeOH crude extract	^**a**^**Sequential extract**			
		
		HEX	DCM	DCM:MeOH	MeOH
^b^**Total alkaloid content (mg BE/g)**					
*Kopsia fruticosa*	100 ± 4.2	63 ± 1.1	129 ± 4.0	99 ± 2.5	46 ± 1.6
*Dyera costulata*	58 ± 2.5	2.4 ± 0.6	11 ± 1.8	68 ± 2.7	41 ± 2.4
*Alstonia angustiloba*	23 ± 0.3	13 ± 2.3	27 ± 3.1	58 ± 1.2	27 ± 1.0
*Calotropis gigantea*	2.7 ± 0.3	3.7 ± 1.0	8.6 ± 1.2	9.6 ± 1.9	9.2 ± 2.7
*Vallaris glabra*	2.7 ± 0.4	4.4 ± 0.8	8.9 ± 0.6	9.2 ± 0.2	8.7 ± 2.2
^b^**Total phenolic content (mg GAE/g)**					
*Dyera costulata*	319 ± 5.5	21 ± 0.2	23 ± 0.1	354 ± 6.0	279 ± 3.0
*Vallaris glabra*	99 ± 3.8	15 ± 0.9	24 ± 0.5	134 ± 1.0	164 ± 13
*Kopsia fruticosa*	83 ± 1.1	39 ± 0.5	20 ± 0.5	129 ± 1.0	84 ± 1.0
*Alstonia angustiloba*	68 ± 2.0	17 ± 0.6	24 ± 0.6	96 ± 1.1	94 ± 1.1
*Calotropis gigantea*	28 ± 0.8	14 ± 0.6	44 ± 1.7	42 ± 0.8	33 ± 0.5
^b^**Radical-scavenging activity (mg AA/g)**					
*Dyera costulata*	377 ± 25	15 ± 0.3	9.3 ± 0.5	349 ± 21	278 ± 5.4
*Vallaris glabra*	84 ± 0.5	6.0 ± 0.3	8.4 ± 0.4	77 ± 2.2	119 ± 6.8
*Kopsia fruticosa*	63 ± 3.2	12 ± 1.4	7.5 ± 0.3	70 ± 2.0	48 ± 1.5
*Alstonia angustiloba*	29 ± 0.9	10 ± 0.2	5.7 ± 0.7	50 ± 1.2	46 ± 2.2
*Calotropis gigantea*	7.6 ± 0.4	5.6 ± 0.3	6.1 ± 0.4	8.0 ± 0.6	14 ± 1.3

^a ^HEX, hexane; DCM, dichloromethane; MeOH, methanol.

^b ^BE, boldine equivalent; GAE, gallic acid equivalent; AA, ascorbic acid. Values are in milligram per gram of extract.

### Phytochemical screening

Phytochemical screening showed the presence of terpenoids in all leaf extracts of *V. glabra *except MeOH extract while saponins and tannins were present in DCM and DCM:MeOH extracts, and in DCM:MeOH and MeOH extracts, respectively (Table 4). Cardenolides were not detected.

**Table 4:** Phytochemical screening of leaf extracts of *Vallaris glabra*

^**a**^**Leaf extract**	^**b**^**Qualitative test**			
	
	^**c**^**Saponin**	^**c**^**Tannin**	^**c**^**Cardenolide**	^**c**^**Terpenoid**
HEX	-	-	-	+++
DCM	+	-	-	++
DCM:MeOH	++	++	-	++
MeOH	-	++	-	-

^a ^HEX, hexane; DCM, dichloromethane; MeOH, methanol.

^b ^Strong (+++), moderate (++) and weak (+) presence, and absent (-). Classification was based on observation of colour intensity and amount of precipitate.

^c ^Saponins, tannins, cardenolides and terpenoids were screened using the frothing, ferric chloride, Kedde and vanillin-H_2_SO_4 _tests.

Terpenoids are used for the treatment of human diseases such as cancer and malaria, and infectious diseases caused by virus and bacterial [39]. Taxol and artimesinin are renowned terpenoid-based anticancer and antimalarial drugs, respectively. Terpenoids inhibit the growth or induce apoptosis of breast cancer cells such as MCF-7, MDA-MB-231 and T47D [40]. They are among the most important natural antimalarial drugs, which also include quinones and alkaloids [41].

Tannins are water-soluble polyphenols that are present in many plant foods [42]. Literature on the effects of tannins on human health is vast and sometimes conflicting. Incidences of esophageal cancer have been attributed to consumption of tannin-rich foods such as herbal teas, suggesting that tannins might be carcinogenic. However, reports have indicated a negative association between consumption of tea and the incidence of cancer. Teas with high tannin content have been suggested to be anticarcinogenic and antimutagenic which may be related to their antioxidative property in protecting cellular oxidative damage against lipid peroxidaton and superoxide radicals. The antimicrobial activities of tannins are well documented.

Saponins are naturally occurring glycosides with a distinctive foaming characteristic and bitter taste [43,44]. They have a wide range of properties, which include both beneficial and detrimental effects on human health. Saponins affect the immune system in ways that help to protect the human body against cancers, and also lower cholesterol levels. They decrease blood lipids, lower cancer risks and lower blood glucose response.

## Conclusions

The DCM extract of *C. gigantea*, and DCM and DCM:MeOH extracts of *V. glabra *inhibited the growth of all six human cancer cell lines. Against MCF-7 and MDA-MB-231 human cell lines, DCM leaf extract of *C. gigantea *had stronger APF activity than standard drugs of xanthorrhizol, curcumin and tamoxifen. With wide spectrum APF activity, leaves of these two species are therefore promising candidates as alternative resources for anticancer drugs. Against K1 strain of *P. falciparum*, all four extracts of *V. glabra *displayed effective APM activity. Selectivity index values suggested that extracts of *V. glabra *are potentially safe for use to treat malaria. The wide spectrum APF and APM activities of *V. glabra *are reported for the first time. This warrants further investigation into its bioactive compounds.

## Competing interests

The authors declare that they have no competing interests.

## Authors' contributions

The study was conducted by SKW as part of her PhD program in Monash University Sunway Campus in Malaysia which is supervised by YYL. Part of the experiments was done in collaboration with Noor Rain and Fariza Juliana from the Institute of Medical Research. SKW analysed the data and drafted the manuscript which was edited and revised by YYL, with comments from counterparts. All authors read and approved the final manuscript.

## Pre-publication history

The pre-publication history for this paper can be accessed here:

http://www.biomedcentral.com/1472-6882/11/3/prepub
